# Apo10 and TKTL1 in blood macrophages as potential biomarkers for early diagnosis of operable breast cancer

**DOI:** 10.1007/s10549-024-07569-3

**Published:** 2024-12-07

**Authors:** Minqing Wu, Qiyu Huang, Lijuan Zhang, Yuying Liu, Musheng Zeng, Chuanbo Xie

**Affiliations:** 1 Cancer Prevention Center, State Key Laboratory of Oncology in South China, Guangdong Provincial Clinical Research Center for Cancer, Sun Yat-Sen University Cancer Center, 651 Dongfengdong Road, Yuexiu District, Guangzhou, 510060 Guangdong China; 2 School of Public Health, Guangxi Medical University, Nanning, 530021 Guangxi China; 3 Department of Breast Oncology, State Key Laboratory of Oncology in South China, Guangdong Provincial Clinical Research Center for Cancer, Sun Yat-Sen University Cancer Center, 651 Dongfengdong Road, Yuexiu District, Guangzhou, 510060 Guangdong China; 4 Department of Experimental Research, State Key Laboratory of Oncology in South China, Guangdong Provincial Clinical Research Center for Cancer, Sun Yat-Sen University Cancer Center, 651 Dongfengdong Road, Yuexiu District, Guangzhou, 510060 Guangdong China; 5 Guangdong-Hong Kong Joint Laboratory for RNA Medicine, Guangzhou, 510060 Guangdong China

**Keywords:** Breast cancer, AUROC, Apo10, TKTL1, CEA, CA-125, CA-199, CA-153

## Abstract

**Objective:**

Blood macrophage Apo10 and TKTL1 detection is a novel, noninvasive cancer screening approach, but its relevance in breast cancer remains uncertain. We compared the potential diagnostic value of Apo10 and TKTL1 with commonly used tumor markers in differentiating breast cancer patients.

**Methods:**

Physical examination and blood sample data from breast cancer patients who did not receive surgery or chemotherapy (retrospective; breast cancer group) and those with benign breast nodules and completely healthy subjects (prospective; control group) were collected from October 2020 to July 2022 at Sun Yat-sen University. Descriptive statistics and receiver operating characteristic (ROC) curves were generated. The area under the ROC curve (AUROC) was calculated to compare the diagnostic efficiency of Apo10 and TKTL1 with conventional biomarkers (carcinoembryonic antigen [CEA], cancer antigens [CA-125, CA-199, CA-153]) in differentiating breast cancer from healthy breasts and benign breast nodules.

**Results:**

From October 2020 to July 2022, 153 breast cancer patients (primarily early-stage disease: n = 113 (73.9%) stage I/II) and 153 control participants (benign breast nodules, n = 56; healthy, n = 97) were included in this study. The breast cancer subtypes were mainly invasive ductal carcinoma (92.8%), with a few cases of DCIS (5.9%), infiltrating lobular carcinoma (0.7%), and mucinous carcinoma (0.7%). Notably, Apo10, TKTL1, and Apo10 + TKTL1 (APT) levels were significantly greater in the cancer group than in the control group (P < 0.001), demonstrating high diagnostic value (AUC = 0.901, 0.871, 0.938) that surpassed CA-125, CA-199, CA-153, and CEA. In a subgroup analysis excluding stage III patients, APT-based breast cancer screening was minimally affected, with the AUROC (0.933–0.938) varying by ≤ 1%.

**Conclusion:**

Compared with conventional biomarkers, Apo10, TKTL1, and APT showed superior early-stage breast cancer screening efficacy, potentially emerging as a promising marker for discriminating breast cancer from healthy breasts and nontumoral lesions.

## Introduction

Breast cancer is one of the most common cancers among women worldwide, with an estimated 2.3 million new cases and 685,000 new deaths in 2020 [1]. From 2000 to 2016, there was a significant increase in age-standardized breast cancer incidence rates among female individuals and an increasing trend in breast cancer mortality rates in China [2]. Therefore, the prevention and treatment of breast cancer are critical targets for the prevention and control of female malignant tumors in China [2]. Early detection and diagnosis are key to the prevention of breast cancer [3]. Although breast cancer has the highest incidence rate among malignancies worldwide, the proportion of early-stage patients remains relatively low. Therefore, early diagnosis is conducive to reducing the burden of the disease.

Breast cancer screening relies primarily on mammography [4], ultrasound [5], and MRI [6]. However, these methods have limitations, such as limited sensitivity in certain cases and high costs. To overcome these challenges, serum tumor markers are emerging as potential screening tools [7]. These markers, which are detectable in blood, offer a noninvasive, potentially sensitive approach for early cancer detection. They can also be used to detect cancer before radiological abnormalities appear, facilitating earlier intervention. Additionally, they complement traditional imaging by providing molecular tumor insights for personalized treatment and disease monitoring. While mammography and imaging remain essential, the use of serum tumor markers represents a promising new screening method.

Despite their widespread use, the standard breast tumor markers carcinoembryonic antigen (CEA), CA-125, CA-199, and CA-153 have limitations in their application for breast cancer diagnosis [8]. CEA, a nonspecific tumor marker, often lacks sensitivity and specificity in breast cancer diagnosis. CA-125, which is primarily associated with ovarian cancer, has limited diagnostic value in breast cancer. Although it may be elevated in some patients with advanced or metastatic breast cancer, its sensitivity and specificity are not high enough for routine diagnosis [9]. Additionally, CA-199 is linked primarily to pancreatic cancer and has limited diagnostic value in breast cancer. Its elevation in breast cancer patients is uncommon and lacks sufficient specificity to serve as a standalone diagnostic tool [10]. Consequently, CA-199 may not be elevated in all patients with breast cancer, and it can be affected by various factors, such as tumor type, stage, and treatment. In addition to lacking sensitivity for early disease detection, CA-153 lacks specificity for breast cancer [11]. These findings indicate that common breast tumor markers have significant limitations in early screening for breast cancer [12]. In light of this, the urgent need to actively pursue and identify novel tumor markers has become paramount [13, 14].

The utilization of flow cytometry to detect Apo10 and TKTL1 in blood macrophages (epitope detection in macrophages, EDIM) is a new method for diagnosing noninvasive cancer [15]. After macrophages engulf tumor cells and their fragments, they leave the tumor and move into the blood through the vascular system [16, 17]. The tumor substances of macrophages are not diluted by the blood but rather become highly concentrated in the cells. This is also a reason for the high sensitivity of EDIM technology and the precise detection and elimination of tumor cells by the innate immune system. Therefore, EDIM detection is better than conventional tumor marker detection, exhibiting superior sensitivity and specificity, enabling earlier detection of tumor signatures and indicating tumor progression stages. Its noninvasive nature and extensive detection range further contribute to its convenience and efficiency as a screening tool [18, 19].

In recent investigations, Apo10 and TKTL1, which are present in blood macrophages, were discovered and subsequently utilized for detecting various types of human cancers [19–22]. Notably, cancer cells proliferate rapidly because they can trigger or possess defective programmed cell death (apoptosis) mechanisms, which Apo10 can detect. Apo10 is an antigenic epitope of DNaseX (deoxyribonuclease) that plays a key role in cell apoptosis [23]. Thus, Apo10 accumulation in cells indicates blocked apoptosis, which can be used as a marker for detecting tumor/proliferative disease formation.

In contrast, TKT is an indispensable enzyme with critical roles in cell metabolism and tumor growth [24]. TKTL1 mainly regulates glycolysis, and its high expression in malignant tumor cells is closely related to tumor invasiveness, therapeutic resistance, and prognosis [24]. TKTL1 also enables tumor cells to rapidly metabolize glucose through glycolysis, generate energy, and discharge large amounts of lactic acid. Thus, the overexpression of TKTL1 provides an acidic environment for tumors to resist immune killing and enhance tumor invasion. Moreover, TKTL1 promotes ribonucleoside synthesis, accelerates DNA replication, and resists free radicals and apoptosis induction therapy [19]. Therefore, high expression of TKTL1 indicates an increased degree of malignancy and poor prognosis [24]. Taken together, these findings indicate that although the Apo10 concentration can usually be detected at the beginning of malignant tumors, the expression of TKTL1 starts to increase only with tumor malignancy and invasiveness. Thus, analyzing these two markers can provide a good illustration of a patient’s malignancy. Research has also demonstrated that the overall sensitivity and specificity of serum Apo10 detection alone for oral squamous cell carcinoma patients, prostate cancer patients, and healthy controls are 92.0% and 94.6%, respectively [19, 23, 25]. The overall sensitivity and specificity of serum TKTL1 levels in distinguishing oral squamous cell carcinoma patients, prostate cancer patients, and healthy controls were 90.6% and 95.9%, respectively. Furthermore, the sensitivity and specificity of the combined detection of serum Apo10 and TKTL1 levels in distinguishing oral squamous cell carcinoma patients, prostate cancer patients, and healthy controls were 95.8% and 97.3%, respectively. Thus, these findings indicate that serum Apo10 and TKTL1 may be promising markers for identifying early-stage tumors [19]. However, the potential of Apo10 and TKTL1 for detecting early breast cancer remain to be further studied.

In a previous study, we established the notable effectiveness of the peripheral blood macrophage factors Apo10 and TKTL1 [22] as screening markers for early lung cancer, exhibiting impressive sensitivity and specificity in differentiating patients from the control group. Building on this foundation, in the present study, we further evaluated the combined detection of Apo10 and TKTL1 for potential use in early breast cancer screening, aiming to distinguish patients with early-stage disease from healthy individuals. We anticipate that these findings will serve as a valuable basis for enhancing the management of early breast cancer patients, ultimately contributing to the avoidance of unnecessary diagnostic procedures and treatments.

## Methods

### Participants and ethics statement

The investigated cohort comprised patients who were diagnosed with breast cancer via pathological biopsy at Sun Yat-sen University Cancer Center (Guangzhou, China) from October 2020 to July 2022. Age-matched controls were selected from people attending their annual physical examination at the Cancer Prevention Center of Sun Yat-sen University Cancer Center. All females meeting the following criteria were included in this study: (1) aged between 20 and 80 years; (2) had no history of immunodeficiency diseases; (3) did not take antirheumatic drugs such as methotrexate, sulfasalazine, hydroxychloroquine, or celecoxib; (4) did not receive immunotherapy or vaccinations 8 weeks before Apo10 and TKTL1 testing; (5) had no prior history of cancer-related surgery; and (6) lacked infectious diseases. The additional inclusion criteria for the benign breast nodule group were as follows: (1) benign BI-RADS-US 2 or 3 breast nodules as determined by at least two senior sonographers via the ACR BI-RADS guidelines and (2) no tumors found in other organs throughout the body. The additional inclusion criteria for breast cancer patients were as follows: (1) no previous history of cancer and the simultaneous presence of other cancers; (2) did not receive surgery or chemotherapy; and (3) no distant metastasis.

### Ethics approval and consent to participate

This study was approved by the institutional review board of Sun Yat-sen University Cancer Center (SYSUCC, ID: G2022-005-01), and all procedures were conducted according to the principles expressed in the Declaration of Helsinki. The authors enrolled the participants via a chart review that was blinded to their personal identifying information (name, address, etc.). Therefore, the rights and welfare of the subjects were not violated in this study. All the participants provided written consent for the anonymous use of their data for their research purposes.

### Apo10 and TKTL1 testing

We collected 2.7 mL of EDTA-anticoagulation venous blood from all the participants 60 min after their last meal. Before testing, the blood samples were stored at room temperature (15–25 °C). The IntraPrep Permeabilization Reagent (Beckman Coulter, Krefeld, Germany) was used for intracellular staining, which was performed with antibodies against CD14 (OFC-14D) and CD16 (Hi-16a). Following permeabilization, two intracellular antibodies, TKTL1 (phycoerythrin [PE]-conjugated, provided by Zyagnum AG, Pfungstadt, Germany) and Apo10 (fluorescein isothiocyanate [FITC], provided by Zyagnum AG, Pfungstadt, Germany), were added. One thousand macrophages (CD14+/CD16+) were selected, and analyses were performed via BD FACSDiva software v8.0 (BD Biosciences, Heidelberg, Germany). Moreover, all the incubations in this protocol were conducted at room temperature in the dark. The EDIM score of the participants was obtained by multiplying the proportion of CD14+/CD16+ cells containing Apo10 and TKTL1 by ten. The EDIM-combined score (APT) was calculated by adding the two scores of Apo10 and TKTL1.

### Measurement of other breast cancer-related biomarkers

Serum tumor markers were prospectively measured just before the start of therapy. CEA was detected via chemiluminescence (Cobas e602, Roche Diagnostics, Germany). CA-125, CA-199, and CA-153 were detected via electrochemical luminescence (Cobas e602, Roche Diagnostics, Germany) using commercial assay kits according to the manufacturer’s instructions. Furthermore, the reference ranges for CEA, CA-125, CA-199, and CA-153 in the serum were 0–5.0 ng/mL, 0–35 U/mL, 0–35 U/mL, and 0–25 U/mL, respectively.

### Statistical analysis

All analyses were performed via R Studio (Version 2023.06.0+421, Copyright © 2022 by Posit Software, PBC) software. Medians and quartiles were calculated to describe the continous variable distributions of Apo10, TKTL1, APT, and other breast cancer-related biomarker levels and percentages were used to describe categorcial varaible distributions. An independent-samples *t* test was used to compare the means between the cancer and noncancer characteristics. We compared the Apo10, TKTL1, and APT levels among the breast cancer patients, the benign breast nodule group, and the true healthy control group via one-way analysis of variance (ANOVA) or Spearman’s rank test. Then, receiver operating characteristic (ROC) curves were generated, and the area under the ROC curve (AUROC) was calculated to compare the diagnostic values of Apo10, TKTL1, APT, and traditional cancer biomarkers in distinguishing breast cancer patients from controls. Intermarker differences in the AUROC were compared via the DeLong test with APT as a control. Apo10, TKTL1, and APT levels were analyzed in breast cancer patients, breast nodule patients, and healthy controls. Differences between groups were compared via t tests, and overall differences were compared via one-way ANOVA. Differences were considered statistically significant at *P* < 0.05.

Considering that patients with stage III breast cancer may exhibit a more severe and complex disease profile, potentially resulting in significant differences in their performance on tumor marker tests compared with patients with early-stage breast cancer or other stages, we conducted sensitivity analyses, which involved reassessing the performance of tumor markers in patients with early-stage or other stages of breast cancer by excluding those with stage III breast cancer. The diagnostic value of APT under different models was subsequently compared in differentiating between breast cancer patients and controls, including healthy and breast nodule groups.

## Results

### Clinical characteristics of the study participants

From October 2020 to July 2022, 153 breast cancer patients at the Department of Breast Oncology Sun Yat-sen University Cancer Center and 153 control participants (benign breast nodules, n = 56; healthy, n = 97) from the Department of Cancer Prevention Sun Yat-sen University Cancer Center were included in this study (Table 1). The average age of the patients in the breast cancer group was 47.96 (± 9.81) years. The breast cancer group included 113 patients with tumors in situ (Tis), stage I or stage II, and 40 patients with stage III disease. The subtypes of the 153 patients included 142 (92.8%) with invasive ductal carcinoma, 7 (5.9%) with DCIS, 1 (0.7%) with infiltrating lobular carcinoma (0.7%), and 1 (0.7%) with mucinous carcinoma. The breast cancer stages of the patients included in this study were determined according to the 8th American Joint Committee on Cancer (AJCC) tumor-node-metastasis (TNM) classification of breast cancer. The average age of the participants in the healthy control group was 48.68 (± 10.27) years (Table 1). No significant difference in the baseline characteristics of age was observed between the breast cancer cohort and the overall control cohort. We found that the Apo10, TKTL1, and APT levels in the breast cancer group were significantly greater than those in the benign breast nodule group and healthy control group (Table 1). Moreover, we observed that the CEA and CA-153 levels in the breast cancer group were greater than those in the benign breast nodule group and healthy control group. However, there were no significant differences in CA-125 or CA-199 across the breast cancer, breast nodule, and healthy control groups (Table 1).

**Table 1:** Comparison of baseline characteristics between patients with breast cancer and controls

Variables	Breast cancer patients (n = 153)	Controls			P value^1^	P value^2^	P value^3^
		Total (n = −153)	Breast nodules (n = 56)	Healthy (n = 97)			
Family history of cancer, n (%)							
No	140 (91.50)	136 (88.89)	49 (87.50)	87 (89.69)	0.034	0.426	0.038
Yes	13 (8.50)	17 (11.11)	7 (12.50)	10 (10.37)			
Age (years), mean (± SD)	47.96 (9.81)	48.68 (10.27)	46.66 (8.46)	49.85 (11.06)	0.532	0.349	0.172
Apo10, M (Q₁, Q₃)	140.00 (137.00, 142.00)	133.00 (130.00, 136.00)	133.00 (128.75, 136.00)	133.00 (130.00, 135.00)	< .001	< .001	< .001
TKTL1, M (Q₁, Q₃)	120.00 (118.00, 123.00)	114.00 (111.00, 117.00)	113.00 (110.75, 117.00)	115.00 (111.00, 117.00)	< .001	< .001	< .001
APT, M (Q₁, Q₃)	261.00 (257.00, 264.00)	248.00 (242.00, 251.00)	246.00 (240.75, 251.25)	248.00 (242.00, 251.00)	< .001	< .001	< .001
CEA, M (Q₁, Q₃)	1.71 (1.10, 2.77)	1.13 (0.79, 1.98)	1.03 (0.71, 1.93)	1.15 (0.81, 1.98)	< .001	< .001	< .001
CA125, M (Q₁, Q₃)	12.20 (9.20, 17.80)	11.50 (8.74, 15.70)	13.10 (9.49, 17.97)	10.90 (8.55, 14.80)	0.281	0.818	0.098
CA153, M (Q₁, Q₃)	11.50 (8.05, 18.90)	9.47 (7.00, 14.10)	9.75 (7.07, 15.20)	9.32 (6.89, 13.00)	< .001	0.045	< .001
CA199, M (Q₁, Q₃)	9.90 (6.68, 18.10)	9.28 (6.06, 14.40)	9.53 (6.68, 16.20)	9.17 (6.04, 13.50)	0.167	0.671	0.105

*M* median, *Q*₁ 1st quartile, *Q*₃ 3rd quartile; P value 1, breast cancer patients versus total controls; P value 2, breast cancer patients versus breast nodule controls; P value 3, breast cancer patients versus healthy controls; APT, addition of the two scores of Apo10 and TKTL1

### Comparison of Apo10, TKTL1, and APT levels in the breast cancer, benign nodule, and control groups

**Fig. 1:**
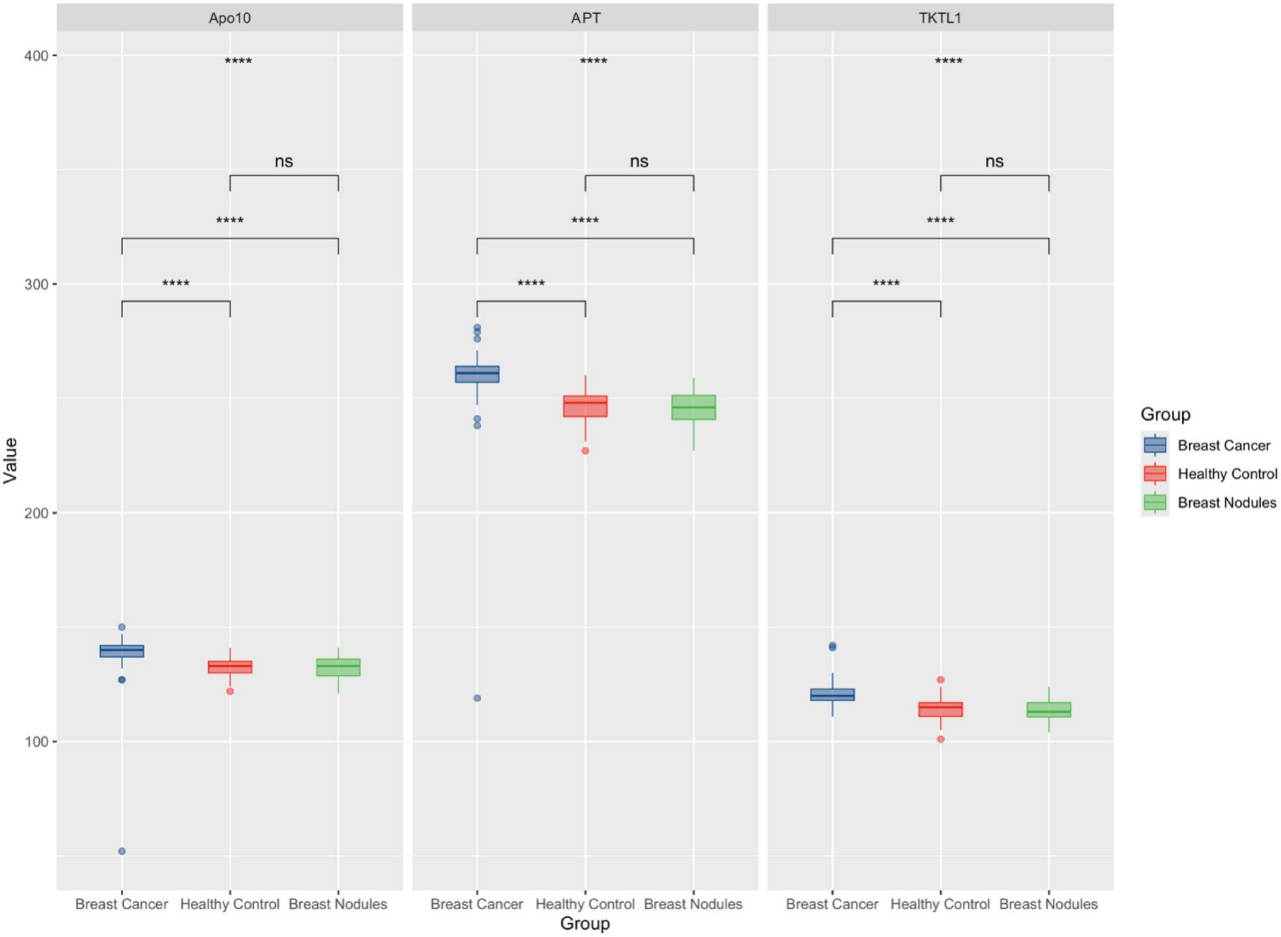
Apo10, TKTL1, and levels across breast cancer patients, breast nodule patients, and healthy controls. *ns* indicates that the difference is not statistically significant; ****indicates that the difference is statistically significant

A detailed comparison of the Apo10, TKTL1, and APT levels in the breast cancer, benign breast nodule, and healthy control groups is presented in Fig. 1. The results indicated that the Apo10, TKTL1, and APT levels in the breast cancer group were consistently and significantly different from those in both the breast nodule group and the healthy control group (P < 0.05). In contrast, no significant difference in those markers was detected between the breast nodule group and those in the healthy control group. On the basis of the determined cutoff values of 136.5, 118.5, and 252.5 for Apo10, TKTL1, and APT (Table 2), respectively, the results revealed that the levels of Apo10, TKTL1, and APT were significantly greater in the breast cancer group than in the overall control cohort (*P* < 0.001), the benign breast nodule group (*P* < 0.001) and the healthy control group (*P* < 0.001).

**Table 2:** Comparison of Apo10, TKTL1, APT, and traditional breast cancer biomarkers in differentiating breast cancer patients from healthy controls

Variables	Cutoff value	Sensitivity	Specificity	AUROC	95% CI		*P*
Apo10	136.500	0.810	0.856	0.901	0.867	0.935	0.002
TKTL1	118.500	0.719	0.869	0.871	0.831	0.910	< 0.001
APT	252.500	0.915	0.830	0.938	0.910	0.965	–
CEA (ng/mL)	1.165	0.725	0.542	0.668	0.609	0.728	< 0.001
CA-125 (U/mL)	19.850	0.229	0.869	0.536	0.471	0.600	< 0.001
CA-153 (U/mL)	17.000	0.301	0.876	0.617	0.554	0.679	< 0.001
CA-199 (U/mL)	15.250	0.314	0.784	0.546	0.481	0.610	< 0.001

*AUROC* area under the receiver operating characteristic curve; *CI* confidence interval; *APT* the addition of the two scores of Apo10 and TKTL1; *P*, comparison of AUROC with other conventional markers using APT as a reference

### Diagnostic value of Apo10, TKTL1 and APT

The diagnostic values of Apo10, TKTL1, and APT were high, with AUCs of 0.901 (95% CI 0.867–0.935), 0.871 (95% CI 0.831–0.910) and 0.938 (95% CI 0.910–0.965), respectively (Table 2). The AUROCs of the other tumor markers (CA-125, CA-199, CA-153, and CEA) for screening for breast cancer were 0.536 (95% CI 0.471–0.600), 0.546 (95% CI 0.481–0.611), 0.617 (95% CI 0.554–0.679), and 0.668 (95% CI 0.609–0.728), respectively (Table 2).

### Diagnostic utility of APT versus commonly used markers in breast cancer

As shown in Table 1, the overall average levels of APT (P < 0.001), CA153 (*P* = 0.005), and CEA (*P* = 0.003) in the breast cancer group were significantly greater than those in the control group. However, no significant difference was observed in CA-125 (*P* = 0.0053) or CA-199 (*P* = 0.440) levels between the two groups.

**Fig. 2:**
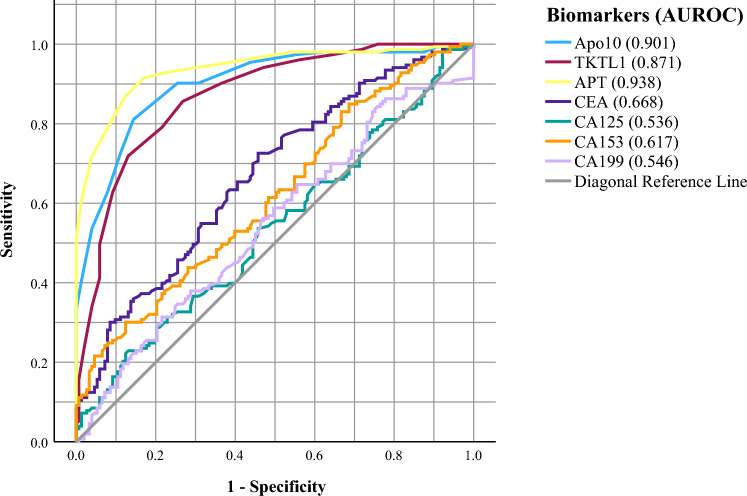
The performance of Apo10, TKTL1, APT, CEA, CA125, CA153, and CA199 in differentiating breast cancer patients from controls. *AUROC* area under the receiver operating characteristic curve; *APT*, the sum of the Apo10 and TKTL1 values

The ROC curve of the diagnostic ability of the five tumor markers (APT, CA-125, CA-199, CA-153, and CEA) for screening for breast cancer was drawn, with the benign breast nodule and healthy control participants as the control group and breast cancer patients as the case group (Table 2, Fig. 2). The results demonstrated that different tumor markers had different diagnostic values for diagnosing breast cancer. The diagnostic value of CA-125 for breast cancer is low, with an AUC of 0.536. Comparatively, the diagnostic values of CA-199, CA-153, and CEA were moderate, with AUROCs of 0.546, 0.617, and 0.668, respectively. In contrast, APT demonstrated the highest diagnostic value, with an AUROC of 0.938. Notably, the individual diagnostic ability of Apo10 (AUROC, 0.901) and TKTL1 (AUROC, 0.871) was also significantly superior to that of commonly used biomarkers (CA-125, CA-199, CA-153, and CEA). Overall, APT was identified as the most promising biomarker for differentiating between breast cancer patients and non-breast cancer patients in this study.

### Sensitivity analysis

**Fig. 3:**
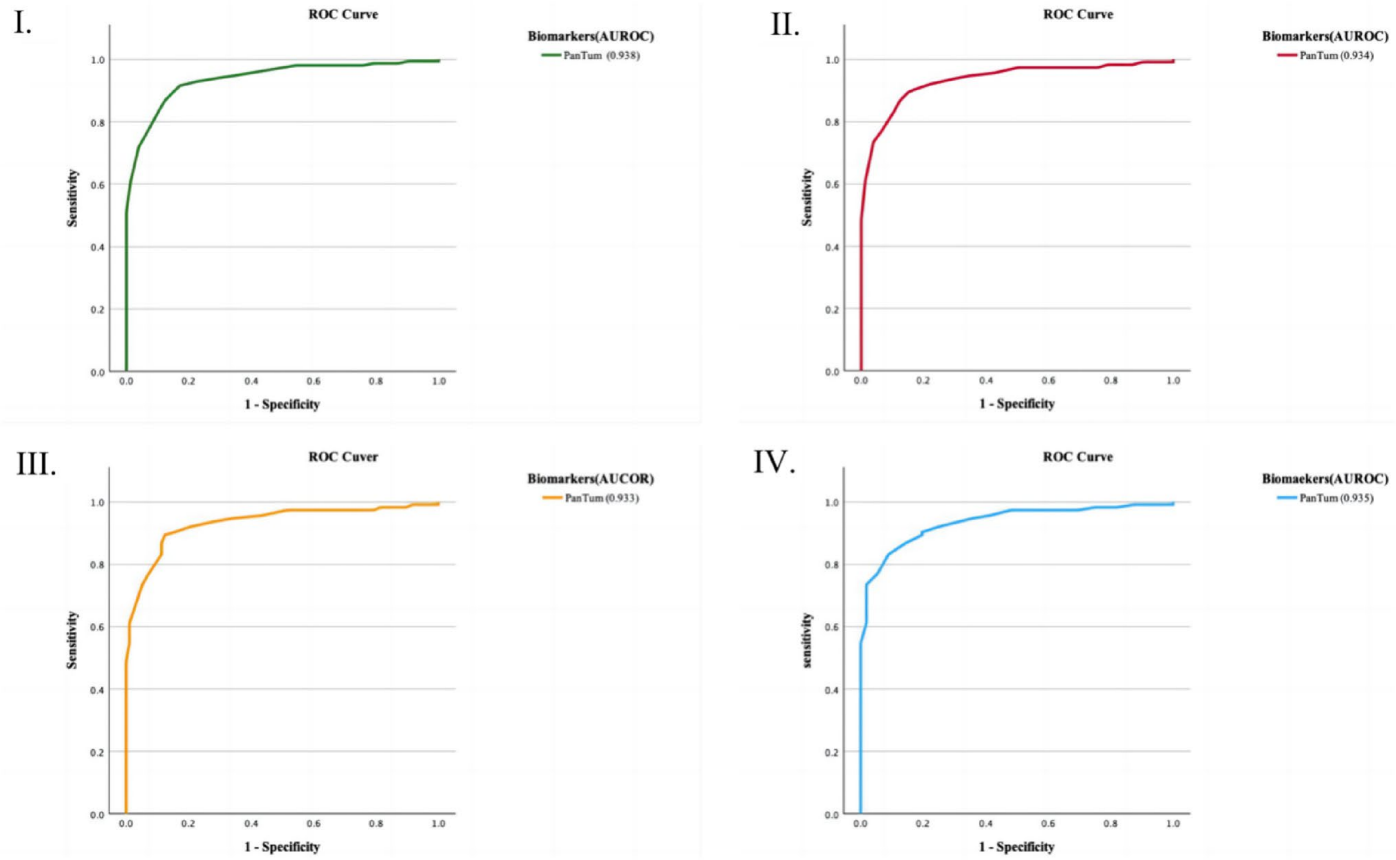
Changes in the performance of APT tumor biomarkers in differentiating breast cancer patients from controls (healthy and nodal groups) after exclusion of patients with stage III breast cancer. **I** Model 1; **II** Model 2; **III** Model 3; **IV** Model 4 (where graphs II, III, and IV are analyses of data after exclusion of patients with Stage III breast cancer). *AUROC* the area under the receiver operating characteristic curve; *APT* the sum of the Apo10 and TKTL1 values

As demonstrated in Fig. 3, the sensitivity analyses excluding patients with stage III breast cancer revealed no significant change in the performance of APT in detecting early-stage breast cancer compared with other stages of breast cancer. From the sensitivity analysis, we observed a difference of less than 1% in the sensitivity of EDIM tumor markers for identifying breast cancer patients after excluding those with stage III breast cancer (Table 3). Furthermore, as show in Table 3, minimal fluctuations in the accuracy disparities, positive predictive values, and negative predictive values among the models were detected. Therefore, this outcome suggests that this tumor marker may exhibit robust generalizability and reliability.

**Table 3:** Comparison of different models of APT tumor biomarkers in differentiating patients with breast cancer from healthy controls via sensitivity analysis

Variables	Cutoff value	Sensitivity	Specificity	Accuracy	PPV	NPV	AUROC	95% CI	
Model 1^a^	252.500	0.915	0.830	0.873	0.843	0.907	0.938	0.910	0.965
Model 2^b^	252.500	0.903	0.830	0.861	0.830	0.920	0.934	0.901	0.966
Model 3^c^	252.500	0.903	0.845	0.876	0.845	0.882	0.933	0.898	0.968
Model 4^d^	252.500	0.903	0.803	0.870	0.804	0.804	0.935	0.899	0.971

*PPV* positive predictive value; *NPV* negative predictive value; *AUROC* area under the receiver operating characteristic curve; *CI* confidence interval

^a^Model 1 includes the original population data;

^b^Model 2 excludes patients with stage III breast cancer;

^c^Model 3 excludes patients with stage III breast cancer and is used to differentiate between breast cancer patients and healthy individuals;

^d^Model 4 excludes patients with stage III breast cancer and is used to differentiate between patients with breast cancer and patients with breast nodules

## Discussion

In this study, our primary objective was to develop an innovative method to improve the detection rate of breast cancer while effectively distinguishing between potentially malignant and nonmalignant breast lesions. These findings revealed that, compared with biomarkers such as CA-125, CA-199, CA-153, and CEA, APT exhibited superior diagnostic value and greater efficacy in distinguishing breast cancer from nonneoplastic breast lesions in patients with breast nodules. Notably, these results were obtained under conditions where baseline characteristics, including age and family history of cancer, were roughly comparable between the breast cancer group and the control group.

Although CEA and CA-153 were also elevated in the blood of patients with breast cancer, APT had greater accuracy than traditional breast cancer biomarkers (i.e., CEA, CA-125, CA-199, and CA-153) in differentiating patients with breast cancer from those with benign breast nodules and healthy controls. The AUROCs of APT, Apo10 and TKTL1 were greater than 87%, whereas the AUROCs of CEA, CA-125, CA-153, and CA-199 were only approximately 60%, indicating the diagnostic significance of APT in differentiating breast cancer from noncancerous conditions. Furthermore, through a comparative analysis of the diagnostic efficacy of APT (Apo10 and TKTL1) with that of conventional markers, we discovered that APT’s sensitivity in detecting breast cancer surpassed that of other traditional tumor markers. Remarkably, the AUROC of the combined APT (0.938) detection approach surpassed the individual performances of Apo10 (0.901) and TKTL1 (0.871). Furthermore, this APT detection method exhibited a reduced false-positive rate, enhancing its diagnostic accuracy.

According to the sensitivity analyses, upon excluding patients with stage III breast cancer, the diagnostic performance of APT across various models exhibited minimal variation. Notably, the change in sensitivity differed by a mere 1.2%, the false positive rate remained stable, and the alteration in AUROC did not exceed 1%. These findings indicate that the diagnostic accuracy, robustness, and efficacy of APT as a diagnostic tool remain largely unaffected by the exclusion of patients with stage III breast cancer. Notably, this underscores the potential for APT to exhibit robust generalizability, delivering consistently reliable diagnostic outcomes across diverse patient cohorts. Furthermore, the diagnostic usefulness of APT for breast cancer are not confined to a particular stage, suggesting its broader applicability to a more comprehensive range of patients.

In the early screening of breast cancer, Apo10 and TKTL1 exhibited unparalleled significance. Apo10 serves as a predictor of tumor formation, whereas TKTL1 is closely correlated with tumor aggressiveness and prognosis. Compared with traditional imaging tests, these biomarkers offer the advantages of earlier tumor lesion detection, enhanced diagnostic accuracy, mitigated treatment costs, and alleviated economic burden. The remarkable innovation lies in the macrophage-based detection experimental system utilized for Apo10 and TKTL1, where the tumor material within macrophages remains undiluted by blood, achieving a high concentration within the cells. As such, this accounts for the exceptional sensitivity of EDIM technology and the ability of the innate immune system to detect and eliminate tumor cells with unparalleled specificity. Consequently, the EDIM surpasses conventional tumor markers, rendering APT a highly sensitive and specific tool for breast cancer screening, outperforming traditional markers.

Some limitations of this study should be noted. First, the number of cases investigated was somewhat limited. However, comparative analysis of patients with benign lesions and healthy controls provided preliminary insights into the potential diagnostic value of the EDIM in breast cancer patients. Second, the accuracy of APT in differentiating different stages of breast cancer remains to be clarified. Third, in this preliminary study, we did not assess the potential application of APT for detecting early cancer relapse or recurrence or predicting DFS and OS, which could be considered a focus of future studies. Fourth, blood levels of Apo10 and TKTL1 serve as pancancer markers and may not be exclusively elevated in patients with breast cancer. Consequently, it is essential to incorporate diagnostic imaging in future studies for a more comprehensive assessment.

We demonstrated that compared with traditional markers, APT has significant specificity and sensitivity for early-stage breast cancer, representing a potential breakthrough in diagnosing and managing breast cancer. The establishment of APT as a superior diagnostic tool deepens our comprehension of the disease and indicates novel avenues for developing more effective screening and treatment methodologies. In the realm of public health, the widespread application of APT-based assays could facilitate earlier detection of breast cancer, leading to improved patient outcomes and ultimately mitigating the overall burden of this debilitating disease.

## Data Availability

The authenticity of this article has been validated by uploading the key raw data onto the Research Data Deposit platform (www.researchdata.org.cn), with the approval RDD number RDDA2024253236.
